# Four *MES* genes from calamondin (*Citrofortunella microcarpa*) regulated citrus bacterial canker resistance through the plant hormone pathway

**DOI:** 10.3389/fpls.2024.1513430

**Published:** 2025-01-20

**Authors:** Yu-Xiong Xiao, Cui Xiao, Zhu Tong, Xiu-Juan He, Ze-Qiong Wang, Hai-Yue Zhang, Wen-Ming Qiu

**Affiliations:** Hubei Key Laboratory of Germplasm Innovation and Utilization of Fruit Trees, Institute of Fruit and Tea, Hubei Academy of Agricultural Sciences, Wuhan, China

**Keywords:** calamondin, citrus bacterial canker (CBC), methylesterase (MES) genes, salicylic acid (SA), jasmonic acid (JA), indole-3-acetic acid (IAA)

## Abstract

Citrus bacterial canker (CBC) disease, caused by *Xanthomonas citri* subsp. *citri* (*Xcc*), is one of the major diseases that seriously endanger citrus production. Citrus regulates the balance of endogenous plant hormones to resist CBC through multiple synthetic pathways, including the demethylation pathways of methyl salicylate (MeSA), methyl jasmonate (MeJA) and methyl indole-3-acetic acid (MeIAA). Here, four methylesterase (MES) genes, *MES1.1*, *MES17.3*, *MES10.2*, and *MES1.5* were screened in the transcriptomes of CBC-resistant and CBC-susceptible varieties after *Xcc* inoculation. Among these *MES* genes, the expression levels of *MES10.2*, *MES1.1*, and *MES1.5* were up-regulated in CBC-resistant varieties, while *MES17.3* was down-regulated in both CBC-resistant and susceptible varieties. Subcellular localization analysis showed that the four MES-encoding proteins were localized in the cytoplasm. Overexpression of *CmMES1.1* and *CmMES1.5* from calamondin (*Citrofortunella microcarpa*) significantly enhanced CBC resistance and increased the salicylic acid (SA) content in calamondin. Conversely, overexpression of *CmMES10.2* and *CmMES17.3* significantly reduced CBC resistance and increased the contents of jasmonic acid (JA) and indole-3-acetic acid (IAA), respectively. We concluded that the resistant varieties confer CBC-resistance by regulating the expression of *CmMES1.1* and *CmMES1.5* to increase SA content, and regulating *CmMES10.2* and *CmMES17.3* to inhibit the synthesis of JA and IAA, respectively. Their ability to regulate the endogenous SA, JA and IAA content through the demethylation pathway was an attractive breeding target for conferring CBC resistance.

## 1 Introduction

Citrus bacterial canker (CBC) is a bacterial disease caused by *Xanthomonas citri* subsp. *citri* (*Xcc*). The pathogen mainly invades citrus plants through wounds or stomas and presents with pustule or cork-like necrotic lesions on young tissues, including leaves, fruit, and stems. In severe cases, symptoms such as leaf drop, branch dieback, and early fruit drop may occur. The appearance and quality of susceptible fruits deteriorate, and yields decrease, causing serious economic losses (Gochez et al., 2020; Das, 2003; Liu et al., 2024). The application of copper bactericides is the main measure for controlling CBC, but it seriously pollutes the environment and affects the quality of citrus (Behlau et al., 2017; Martinez et al., 2016). At present, *Xcc* can infect most citrus cultivars, such as lime (*Citrus aurantifolia*), sweet orange (*C. sinensis*), and grapefruit (*C. paradisi*). However, ‘Meiwa’ kumquat (*Fortunella crassifolia*), ‘Marumi’ kumquat (*F. japonica*), ‘Nagami’ kumquat (*F. margarita*) and calamondin (*Citrofortunella microcarpa*) are less susceptible to the disease (Ference et al., 2020; Long et al., 2019; Duan et al., 2022). Intensive studies on the mechanism differences of citrus varieties in response to CBC will provide a theoretical basis and contribute to the improvement of CBC resistance breeding.

Plant hormones are closely related to the pathogenesis of CBC in citrus. Salicylic acid (SA) played a positive role in resistance to CBC, exogenous treatments with SA increase the resistance of CBC in susceptible citrus cultivars (Wang and Liu, 2012). It is noteworthy that overexpression of the *Arabidopsis NPR1* increased the CBC resistance of susceptible citrus cultivars (Zhang et al., 2020), and most recent studies showed the *NPR1-like* genes, as receptors for SA, could be stimulated by *Xcc* infection, implying their responsiveness to CBC challenges (Ali et al., 2024). In addition, SA could regulate plant resistance through antagonism and synergism with other plant hormones (Robert-Seilaniantz et al., 2011). For example, JA could antagonize SA-mediated immune responses, promoting plant susceptibility to pathogens (Yang et al., 2017). Similarly, MeJA treatment resulted in CBC susceptibility, and SA treatment significantly enhanced the CBC resistance in Wanjincheng orange (*C. sinensis*). However, the accumulated JA inhibited effective SA-mediated defense and promoted CBC symptom formation (Long et al., 2019). Furthermore, auxin promoted CBC susceptibility in citrus, while the inhibitor of gibberellin synthesis, chlorocholine chloride (CCC), antagonized auxin signaling and inhibited CBC symptom formation (Cernadas and Benedetti, 2009).

The demethylation pathway of plant hormones played an important role in plant immune response, the demethylation of the methyl esters of IAA, SA, and JA was catalyzed by methylesteras (MES), which belonged to the α/β hydrolase superfamily (Nardini and Dijkstra, 1999). SABP2 (salicylic acid binding protein 2), a tobacco methyl salicylate (MeSA) esterase, was essential for the development of systemic acquired resistance (SAR) (Kumar and Klessig, 2003; Forouhar et al., 2005; Gong et al., 2023). Previous studies showed that the common beans *PvMES1*, soybean *GmSABP2-1*, potato *StMES1* and *Lycium chinense LcSABP* also had salicylate methyl esterase activity, which could convert methyl salicylate to SA and participate in stress response (Xue et al., 2021; Lin et al., 2024; Manosalva et al., 2010; Li et al., 2019). In *Arabidopsis thaliana* genome, twenty MESs homologous to SABP2 were identified by protein homology analysis and named AtMES1-AtMES20 (Yang et al., 2008). *AtMES1/7/9* were induced during pathogen infection, and overexpression of *AtMES1/7/9* in SABP2-silenced tobacco could restore SAR deficiency while silencing them could lead to MeSA accumulation (Vlot et al., 2008; Gao et al., 2021). Similarly, *MES* family genes from *Brassica oleracea* var. *Capitata* actively responded to *Plasmodiophora brassicae* infection (Manoharan et al., 2016). *FvMES2* from strawberry (*Fragaria vesca*) were involved in MeSA demethylation and responded significantly to *Botrytis cinerea* stress through the SA signaling pathway (Jia et al., 2024). Furthermore, some MES proteins showed specificity and preference to the specific substrate, while some MES proteins shared multiple methylesterase activity. The substrate specificity test of AtMES proteins showed that five, six and eight AtMES proteins had salicylate methyl esterase activity, jasmonate methyl esterase activity and methyl IAA esterase activity, respectively (Yang et al., 2008; Vlot et al., 2008). AtMES17 had only IAA methylesterase activity, while AtMES1 had higher SA methylesterase activity and lower JA and IAA methylesterase activity (Yang et al., 2008; Vlot et al., 2008). VvMES5 from grape (*Vitis Vinifera*) showed similar jasmonate methyl esterase activity as its homologue AtMES10 (Zhao et al., 2016). PpMES1 from peach (*Prunus persica* L. Batsch) only had methyl jasmonate esterase activity, while PpMES2 had methyl jasmonate and salicylate methyl esterase activity (Cao et al., 2019). CsMES1 from sweet orange (*C. sinensis*), a homologue of tobacco SABP2, could convert MeSA to SA, and its inhibitor promoted CBC symptom formation, suggesting that CsMES1 might play a positive role in CBC resistance (Lima Silva et al., 2019). However, the activity of most MES family genes and their function in CBC remain unclear.

To understand the function of *MES* family genes responded to CBC in citrus, four *MES* family genes were obtained by comparing the transcriptomes of CBC- resistant and CBC-susceptible varieties. The cytoplasmic localization of the four candidate genes from calamondin was determined through the transient transformation of *Nicotiana benthamiana* leaves. The results of *Xcc* inoculation and plant hormones determination of transiently overexpressing calamondin and ‘Taoyecheng’ sweet orange leaves showed that overexpression of *CmMES1.1* and *CmMES1.5* enhanced CBC resistance and increased SA content, while overexpression of *CmMES17.3* and *CmMES10.2* promoted citrus canker disease development and increased auxin and jasmonic acid content, respectively. These *MES* genes might be important genetic resources for screening and breeding CBC-resistant varieties.

## 2 Materials and methods

### 2.1 Plant and bacterial materials

The calamondin and ‘Taoyecheng’ sweet orange were grown in a greenhouse at 25 ± 1°C in Wuhan, China. The leaves of calamondin and ‘Taoyecheng’ sweet orange were utilized in *Xcc* inoculation experiments and transient overexpression analyses. The *Xcc* strain was routinely cultured at 28°C in an Lysogeny Broth (LB) solid culture medium.

### 2.2 Transcriptome analysis of citrus after *Xcc* inoculation

The transcriptome data of CBC-resistant ‘Meiwa’ kumquat (*F. crassifolia*) and CBC-susceptible ‘Mexican’ lime (*C. aurantifolia*) responding to *Xcc* infection at 24 hpi, 48 hpi and 72 hpi were obtained through CitrusKB database (http://bioinfo.deinfo.uepg.br/citrus/) (**Supplementary Table S1**).

In addition, the young leaves of calamondin were selected for *Xcc in vivo* inoculation. *Xcc* was cultured overnight at 28 °C. The activated bacterial solution was diluted to OD=0.6 (10^8^ cfu/ml) with sterile water and further diluted to 10^4^ cfu/ml for infiltration inoculation. Sterile water inoculation was used as a blank control. Subsequently, leaf samples were collected at different time points 1 dpi, 3 dpi and 5 dpi for transcriptome analysis and differentially expressed genes (DEGs) analysis. (**Supplementary Table S2**).

### 2.3 Protein homology, gene structure and promoter element analyses

The whole MES proteins of ‘Hong Kong’ kumquat (*F. hindsii*) were downloaded from the CPBD database (http://citrus.hzau.edu.cn/index.php, accessed on 13 May 2024), and 20 MES proteins of *A. thaliana* were obtained from the TAIR database (https://www.arabidopsis.org/, accessed on 13 May 2024). Four candidate MES proteins from calamondin were cloned and sequenced; the primers were listed in **Supplementary Table S3**. Then, the phylogenetic relationships of the MES proteins were constructed using the neighbor-joining method by MEGA 11.0 software with the following parameters: Poisson model, pairwise deletion, and 1000 bootstrap replications. The phylogenetic trees were visualized using the iTOL web package. All the gene accession numbers and protein sequences are listed in **Supplementary Tables S4**.

The structure of MES proteins from *F. hindsii* was analyzed using the MEME Suite 5.5.2 online program (Multiple Em for Motif Elicitation 5.5.2, http://alternate.meme-suite.org/tools/meme, accessed on) and TBtools (https://github.com/CJChen/TBtools, accessed on). The 2000-bp promoter sequences of the upstream regions of MES proteins were obtained. Cis-acting elements were predicted via PlantCARE (http://bioinformatics.psb.ugent.be/webtools/plantcare/html/, accessed on), and the responsive regulatory elements were analyzed via TBtools.

### 2.4 Analysis of tissue specific and plant hormone treatment

Due to CBC mainly infects young leaves, stems and fruits of citrus, young and mature leaves, stem and fruit were used for tissue-specific analysis of four methylesterase family genes.

Young leaves of calamondin were selected for plant hormone response analysis. 500 µM SA, 100 mg/L IAA, 5 mg/L GA_3_, 200µM mg/L methyl Jasmonate, and 100 mM abscisic acid (ABA) were used to spray the calamondin leaves. Hormone-treated leaf samples were collected 1, 2, and 3 days after spraying, and untreated leaf samples were used as a control.

### 2.5 Quantitative real-time PCR

The total RNA of calamondin leaves was extracted using the RNA prep Pure Plant Kit (Tiangen, Beijing, China), and the cDNA was obtained by the Monad MonScriptTMRTIII Super Mix with dsDNase (Two-Step) (Monad, Suzhou, China). The Monad MonAmpTM SYBR^®^ Green qPCR Mix (Vazyme, Nanjing, China) and Applied Biosystems PCR-7500 (ABI, USA) were used for amplification and the qRT-PCR analysis. The primers used for the qRT-PCR are shown in **Supplementary Table S3**. Three biological replicates were conducted.

### 2.6 Subcellular localization analysis

The CDS sequences of *CmMES1.1* (441 bp), *CmMES17.3* (885 bp), *CmMES10.2* (642 bp) and *CmMES1.5* (804 bp) from calamondin were inserted into the PK7203-RFP vector to generate a fusion protein of the target gene with RFP. First, PK7203-RFP vector was digested by Sall-HF enzyme, and then the coding sequence of each MES gene was cloned into the vector by homologous recombination cloning technology. The overexpression vector includes an enhanced green fluorescent protein (EGFP) activated by minimal CaMV 35S promoter as a screening marker, and an MES protein fusion mRFP promoted by minimal CaMV 35S promoter for subcellular localization. Please refer to **Supplementary Figure S1** for detailed map. The specific steps were as follows: first, the single colony of each *Agrobacterium* strain EHA105 was cultured on LB medium at 28 °C overnight; then resuspended with infiltration buffer (10 mM 2-(N-morpholino) ethanesulfonic acid, pH 5.85; 10 mM MgCl_2_; 30 mg/L acetosyringone) to OD=0.6; finally the infiltration buffer was injected into the leaves of *Nicotiana benthamiana*, which was then cultured in the dark for 12 hours and 16-h/8-h light/dark photocycle for 2 days. The fluorescence was observed through a laser scanning confocal microscope (TCS-SP8 SR, Leica, Wetzlar, Germany). The primers used for the construction of the overexpression vector are shown in **Supplementary Table S3**.

### 2.7 Transient expression of citrus leaves

The above four EHA105 strains for subcellular localization analysis were used in transient transformation assay, with the leaves of calamondin and ‘Taoyecheng’ sweet orange (*C.sinensis*) as explants. The optimized transient transformation method was used to obtain overexpressed calamondin leaves. The specific steps were as follows: first, the single colony of each *Agrobacterium* strain EHA105 was grown at 28 °C overnight; then resuspended with infiltration buffer (10 mM 2-(N-morpholino) ethanesulfonic acid, pH 5.85; 10 mM MgCl_2_; 30 mg/L acetosyringone) to OD=1.0-1.5; finally injected the infiltration buffer into the young calamondin leaves and ‘Taoyecheng’ sweet orange with a needleless syringe, and injected repeatedly for three times at one-hour intervals and cultured in the dark for 2 days. Finally, the instantaneous transformation of *Agrobacterium* was determined by green fluorescence observation (**Supplementary Figures S2**).

### 2.8 CBC resistance analyses of the MESs

To determine the CBC resistance ability of the four key MES genes, leaves were picked from the transiently transformed calamondin and ‘Taoyecheng’ sweet orange leaves for *in vitro Xcc* inoculation. 0.1 mL *Xcc* (10^8^ cfu/ml) was infiltrated into the calamondin leaves, and 5 µL *Xcc* (10^8^ cfu/ml) was dripped onto each puncture site made with a pin (0.5 mm in diameter) on’Taoyecheng’sweet orange leaves. The leaves were then cultured in an incubator at 28°C, with 80% relative humidity and a 16-h/8-h light/dark photocycle. CBC symptom and resistance degree were evaluated at 5 and 15 days post-inoculation (dpi) of calamondin and ‘Taoyecheng’ sweet orange leaves, respectively. Lesion area was analyzed by ImageJ software, and all experiments were repeated at least three times.

### 2.9 Plant hormone determination

Approximately 100 mg of frozen calamondin leaves were ground and extracted with 1 ml ice-cold 50% aqueous acetonitrile (vol/vol). Subsequently, the samples were sonicated for 3 min and extracted for 30 min at 4°C. The supernatant was obtained after centrifugation (10 min, 12,000 rpm, 4°C) and purified using C18 reversed-phase. After this solid phase extraction, the samples were blown dry by nitrogen and dissolved in 200 μl of 30% acetonitrile (vol/vol). Ultra-efficient liquid chromatography (Vanquish, UPLC, Thermo, USA) and high-resolution mass spectrometry (Q Exactive, Thermo, USA) were used to determine the plant hormone levels. The analytical conditions were as follows: column: Waters HSS T3 (50×2.1 mm, 1.8 μm); mobile phase: phase A is ultra-pure water (containing 0.1% acetic acid) and Phase B is acetonitrile (containing 0.1% acetic acid). The data were collected using the Q Exactive high-resolution mass spectrometry system from Thermo Fisher Scientific and processed using TraceFinder Software. Triplicates were conducted for determine each plant hormone.

### 2.10 Statistical analysis

All data analyses were performed using GraphPad Prism 6.0 (GraphPad, USA), the results were presented as means ± standard deviation (SD), and comparisons were made using ANOVAs with Duncan’s multiple range test.

## 3 Results

### 3.1 Expression pattern analyses of *MES* family genes in response to *Xcc* infection

**Figure 1:**
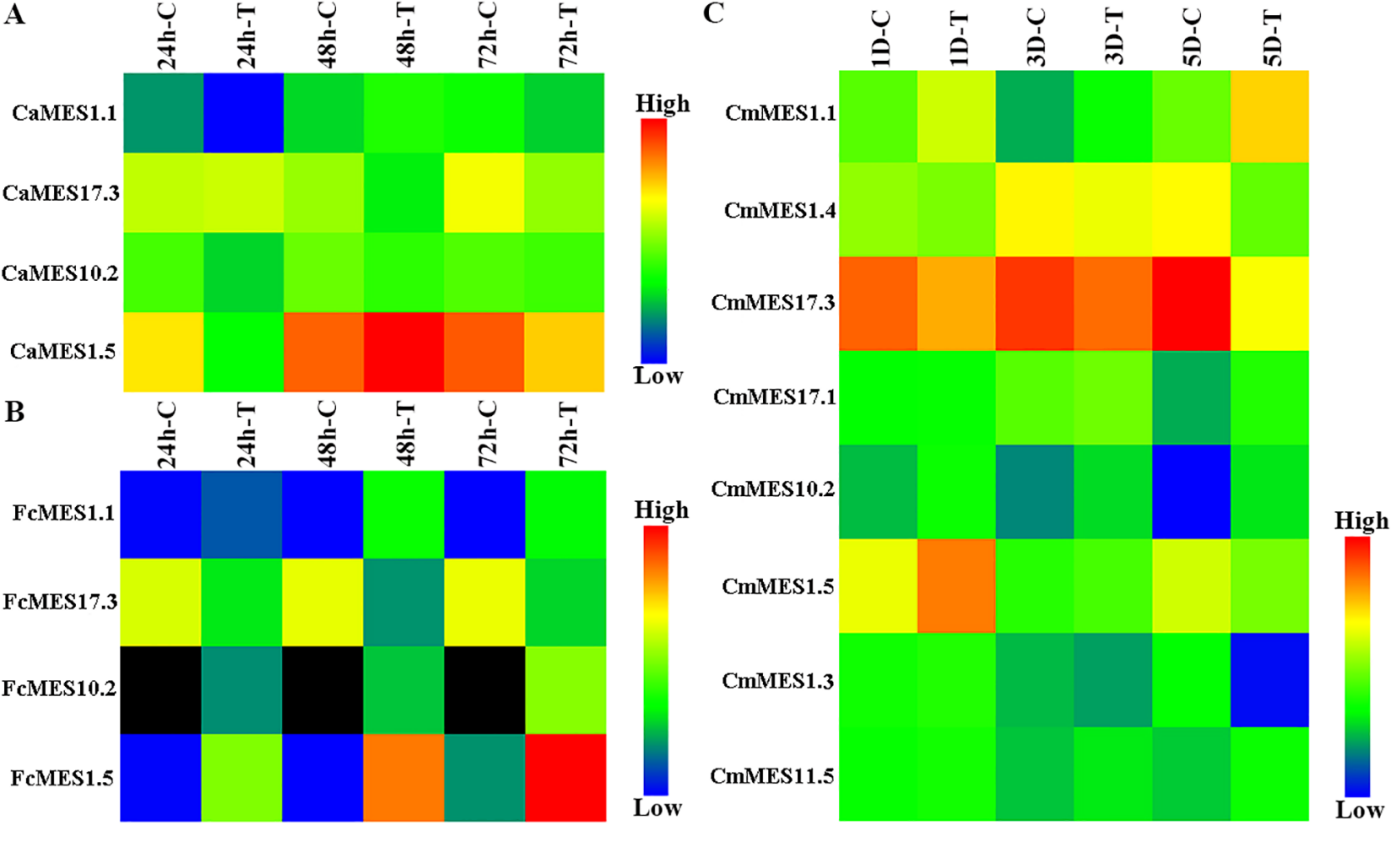
Expression heatmap of *MES* family genes after *Xcc* infection. **(A)** ‘Mexican’ lime (*C. aurantifolia*). **(B)** ‘Meiwa’ kumquat (*F. crassifolia*). **(C)** Calamondin (*Citrofortunella microcarpa*). hpi, hour post-inoculation; dpi, day post-inoculation; C, control; T, treatment. Black indicates no detection.

The transcriptome data of CBC-resistant ‘Meiwa’ kumquat (*F. crassifolia*) and calamondin (*Citrofortunella microcarpa*), and CBC-susceptible ‘Mexican’ lime (*C. aurantifolia*) in response to *Xcc* infection were re-analyzed with Tbtools. Eight differentially expressed *MES* genes were screened from CBC-resistant calamondin, including *MES1.1*, *MES1.4*, *MES17.3*, *MES17.1*, *MES10.2*, *MES1.5*, *MES1.3* and *MES11.5*. Among them, *MES1.1*, *MES17.3*, *MES10.2* and *MES1.5* were shared by CBC-resistant ‘Meiwa’ kumquat and calamondin and CBC-susceptible ‘Mexican’ lime. Compared with the control, the expression of *MES1.1*, *MES10.2* and *MES1.5* were down-regulated at 24 hpi after *Xcc* inoculation in CBC-susceptible ‘Mexican’ lime, but up-regulated at 24 hpi hpi, 48 hpi and 72 hpi after *Xcc* inoculation in CBC-resistant ‘Meiwa’ kumquat and up-regulated at 1 dpi and/or 3 dpi and/or 5dpi after *Xcc* inoculation in CBC-resistant calamondin. The expression of *MES17.3* was down-regulated in all three citrus species. The expression level of *MES17.3* was decreased by 7.5 and 3.1 times at 48 hpi and 72 hpi after *Xcc* inoculation in CBC-susceptible ‘Mexican’ lime, respectively (**Figure 1A**). However, it was decreased by 16.8, 54.2 and 26.9 times at 24 hpi, 48 hpi and 72 hpi in CBC-resistant ‘Meiwa’ kumquat and 1.8, 1.5 and 8.5 times at 1 dpi, 3 dpi and 5 dpi in CBC-resistant calamondin (**Figures 1B, C**). These results indicated that *MES* family genes played an important role in CBC resistance, among which *MES1.1*, *MES17.3*, *MES10.2* and *MES1.5* might be key regulatory genes.

### 3.2 qRT-PCR verification of *MES* genes in response to *Xcc* infection

**Figure 2:**
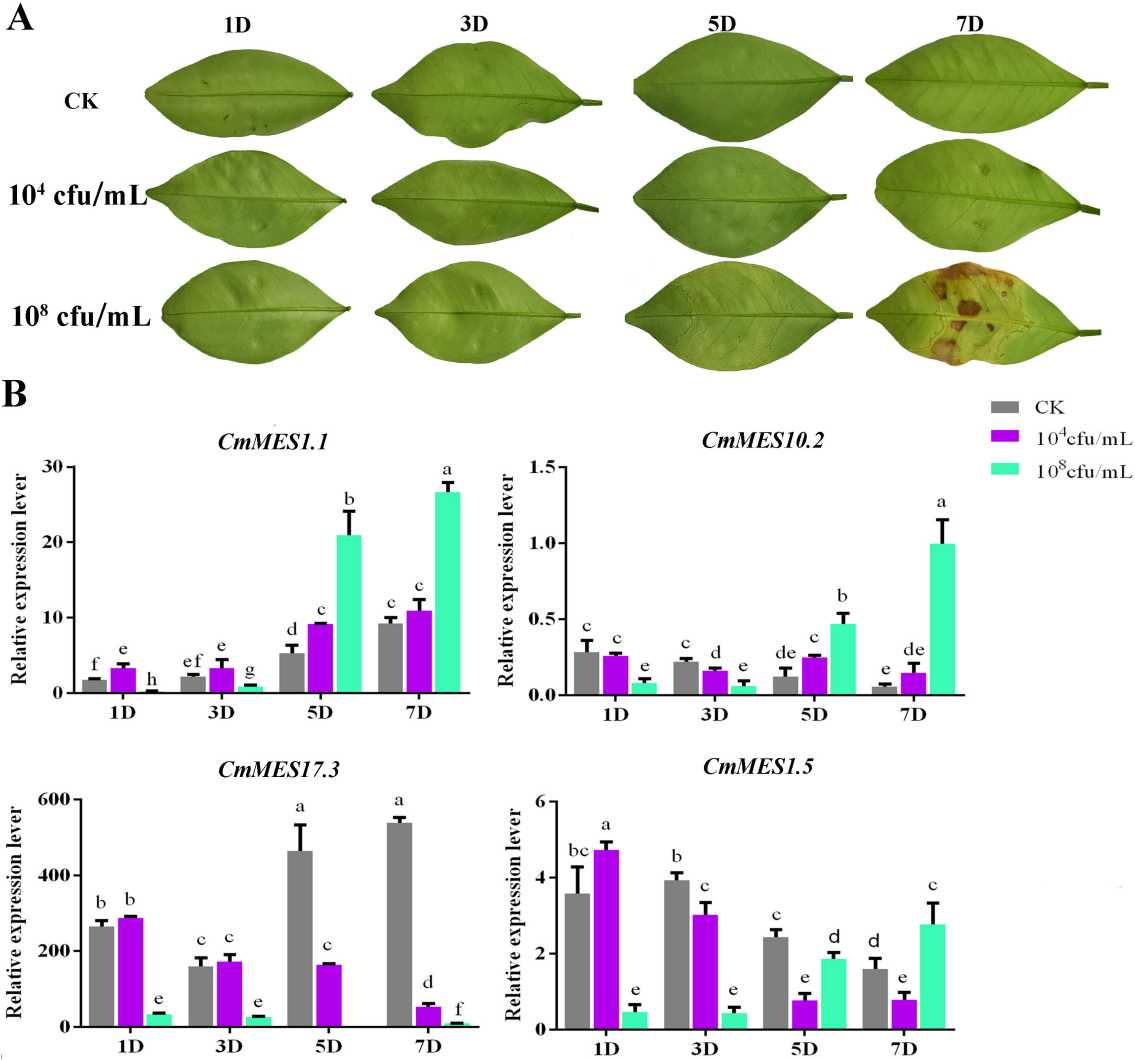
The response of four key *MES* genes upon *Xcc* infection. **(A)** Phenotypic observation of young leaves of calamondin inoculated with low (10^4^ cfu/ml) and high (10^8^ cfu/ml) concentrations of *Xcc*. **(B)** The expression patterns of four key MES genes inoculated with low and high concentrations of *Xcc* (*p* < 0.05, ANOVAs with Tukey’s multiple range test).

The expression patterns of four *MES* genes (*CmMES1.1*, *CmMES17.3*, *CmMES10.2*, and *CmMES1.5*) that responded to *Xcc* were further analyzed by qRT-PCR in calamondin. Young leaves of calamondin were infiltrated with low (10^4^ cfu/ml) and high (10^8^ cfu/ml) concentrations of *Xcc* suspension and analyzed at four stages (1 dpi, 3 dpi, 5 dpi, and 7 dpi) after *Xcc* inoculation. Compared with sterile water inoculation used as a blank control, the leaves of calamondin inoculated with a low concentration of *Xcc* showed no obvious symptoms within 7 dpi, while the leaves inoculated with a high concentration of *Xcc* had obvious pustule symptoms after 5 dpi and appeared hypersensitive necrotic after 7 dpi (**Figure 2A**). When inoculated with a high concentration of *Xcc*, the expression levels of *CmMES1.1*, *CmMES10.2* and *CmMES1.5* were all significantly down-regulated at an early stage (1 dpi and 3 dpi) and up-regulated at a later stage (5 dpi and/or 7 dpi) (**Figure 2B**). However, the expression level of *CmMES17.3* was significantly down-regulated at the whole stage, particularly at the later stage (**Figure 2B**). The expression levels of the four MES genes were only slightly regulated by low concentration *Xcc* infection. For example, all the four *MES* genes were slightly up or down regulated at the early stage (1 dpi and 3 dpi). Although *CmMES1.1* and *CmMES10.2* were significantly up-regulated at 5 dpi, *CmMES17.3* and *CmMES1.5* were significantly down regulated at 5 dpi and 7 dpi, their up-regulation and down-regulation levels were significantly lower than those with high concentration *Xcc* infection. These results indicated that the function of these *MES* genes might be strongly activated at the later stage upon *Xcc* infection associated with the development of the symptoms.

### 3.3 Protein homology, gene structure, and promoter element analysis of *MES* genes in citrus

**Figure 3:**
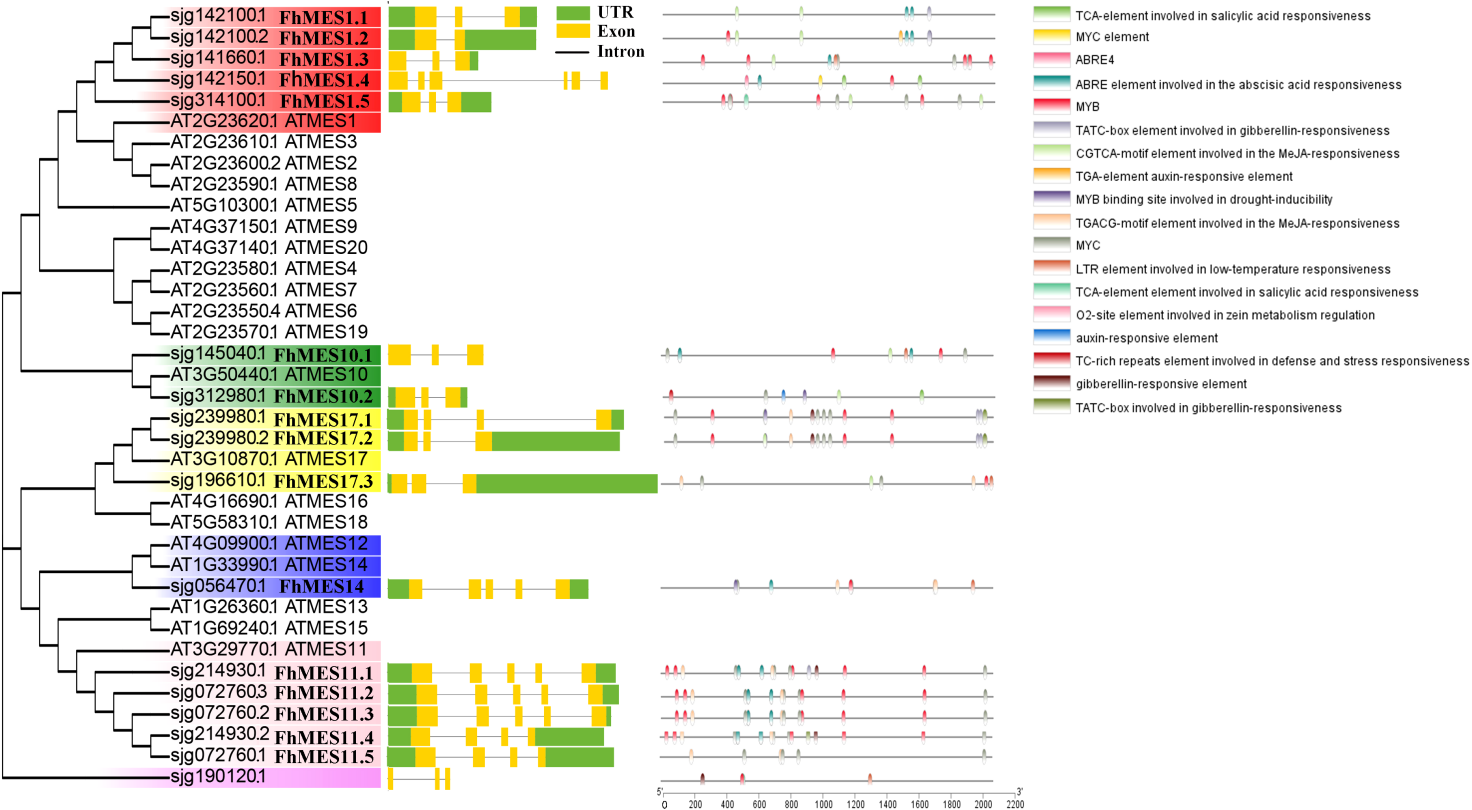
The phylogenetic relationship, gene structure and promoter element analysis of the MES family proteins in kumquat. Conserved MES proteins from kumquat and *A. thaliana* were aligned using the ClustalW function of MEGA11. The phylogenetic tree (with 1000 replicates) was constructed by NJ method and bootstrapping analysis. Different colors represent different types of MES proteins in kumquat. Gene structure of *MES* genes in kumquat. The yellow bar indicates the coding sequence (CDS), the line indicates the intron, and the green bar indicates the untranslated region (UTRs). The cis-acting element within the 2000-bp upstream sequence of the kumquat *MES* gene. This study used the database PlantCARE to predict the motif, different colors represent different promoter elements.

To explore the phylogenetic relationships of kumquat MES proteins, we constructed a phylogenetic tree based on the amino acid sequences of 12 kumquat MES proteins and 20 A*.thaliana* MES proteins. In the kumquat (*F. hindsii*) genome, *Sjg142100, Sjg239980*, and *Sjg214930* contained two spliceosomes, *Sjg072760* had three spliceosomes, and the rest had only one spliceosome. Based on comparison analysis with *A.thaliana* MES proteins, the kumquat *MES* family genes were mainly divided into six groups, namely group *AtMES1* (*FhMES1.1*-*FhMES1.5*), group *AtMES10* (*FhMES10.1* and *FhMES10.2*), group *AtMES11* (*FhMES11.1-FhMES11.5*), group *AtMES14* (*FhMES14*), group *AtMES17* (*FhMES17.1*-*FhMES17.3*) and group *Sjg190120* (**Figure 3**). In addition, four candidate CBC resistance-related *MES* genes from calamondin were cloned and sequenced, and they belonged to *FhMES1.1*, *FhMES17.3*, *FhMES10.2* and *FhMES1.5* (**Supplementary Figure S3**). Gene structure analysis showed that the kumquat *MES* family genes contained 2 - 6 exons. Promoter element analysis showed that the promoters of kumquat *MES* family genes contained a large number of plant hormone-responsive elements (SA, auxin, methyl jasmonate, ABA (abscisic acid), and gibberellin), stress (low temperature and drought) and defense-related response elements (**Figure 3**).

Therefore, the *MES* genes might be involved in response of plant hormones including SA, IAA, JA, ABA or GA.

### 3.4 Tissue-specific expression and plant hormone responses of *MES* genes

**Figure 4:**
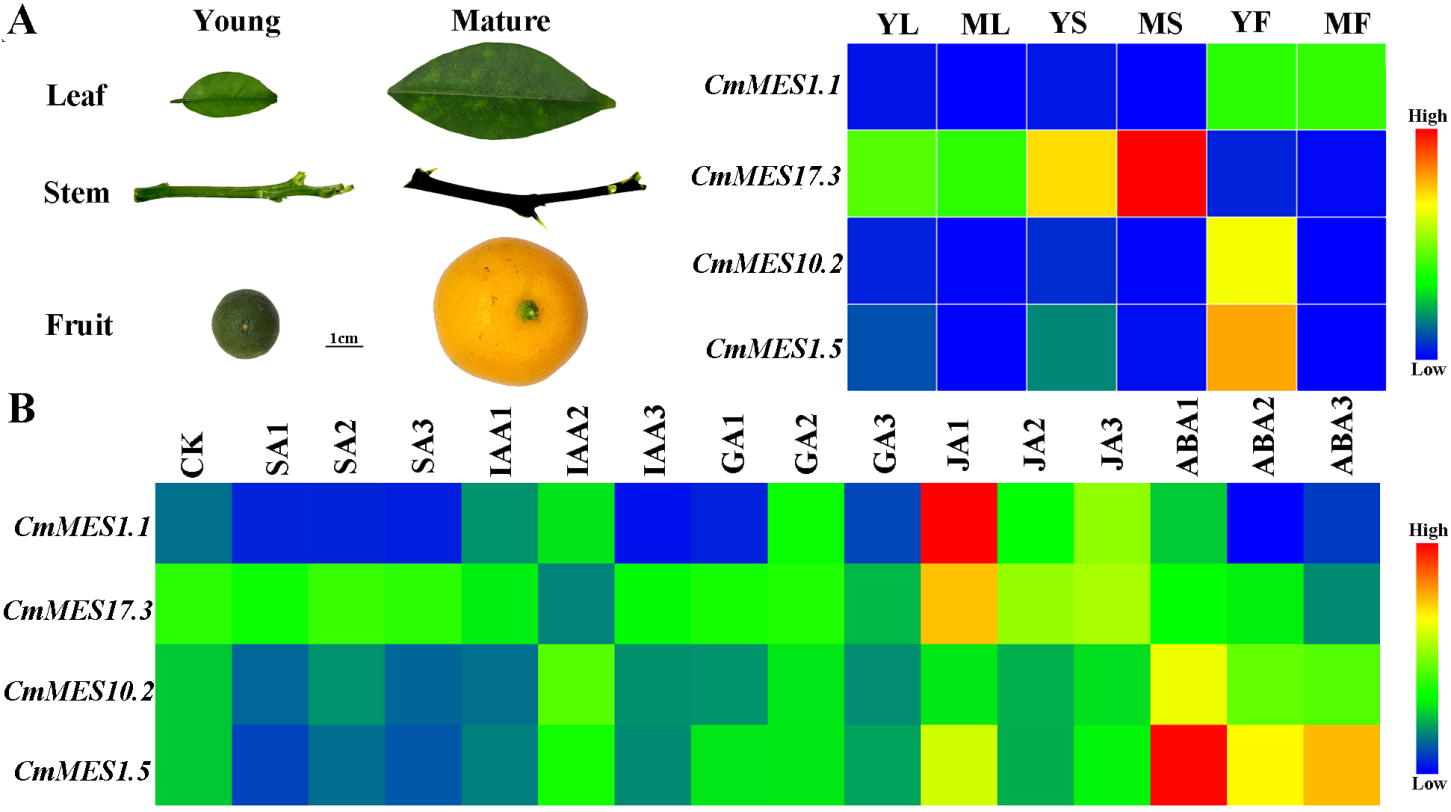
The expression patterns of four calamondin *MES* genes in different tissues and in response to different hormone treatments. **(A)** The tissue-specific expression patterns of four *MES* genes. YL, Young leaf; ML, Mature leaf; YS, Young stem; MS, Mature stem; YF, Young fruit; MF, Mature fruit. **(B)** The expression patterns of four *MES* genes at 1, 2, and 3 days after different plant hormone treatments. CK, samples untreated for 0 days; SA, 500µM SA treatment; IAA, 100 mg/L indole-3-acetic acid treatment; GA, 5 mg/L GA3 treatment; JA, 200µM methyl Jasmonate treatment; ABA, 100 mM abscisic acid treatment.

The stems, leaves, and fruits of calamondin at different growth stages were used to analyze the tissue-specific expression patterns of *CmMES1.1, CmMES17.3, CmMES10.2*, and *CmMES1.5*. The results indicated that *CmMES1.1* was mainly expressed in fruits. However, the expression level was higher in the young tissues of leaves and stems than in mature tissues. *CmMES17.3* was mainly expressed in stems, especially in mature stems. *CmMES10.2* and *CmMES1.5* were highly expressed in young tissues, particularly in young fruits (**Figure 4A**). Furthermore, the four *MES* genes were regulated by different plant hormones. *CmMES1.1* and *CmMES1.5* were down-regulated by SA treatment and up-regulated by JA treatment, meanwhile, *CmMES1.5* was also significantly up-regulated by ABA treatment. *CmMES17.3* was mainly up-regulated by JA treatment and *CmMES10.2* was mainly up-regulated by ABA treatment (**Figure 4B**). These results revealed that the four *MES* genes might prone to express in young tissues and be involved in different plant hormone pathways.

### 3.5 Subcellular localization analysis of the four MES proteins

**Figure 5:**
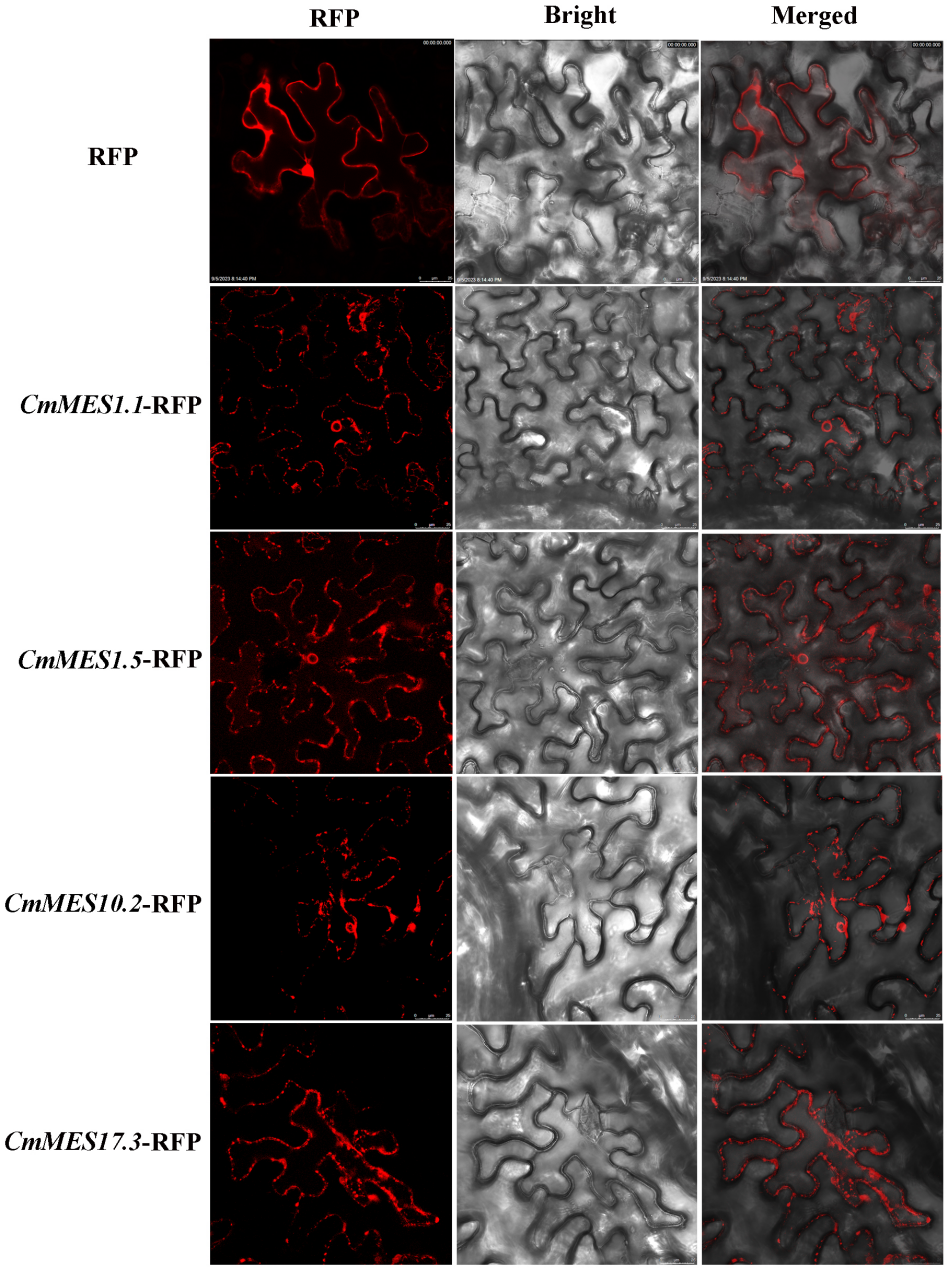
Subcellular localization of four calamondin *MES* genes in *N. benthamiana* leaves. Fields of view are shown as fluorescence field, bright field, and merged images, scale bar = 25 μm.

To examine the subcellular localization of the four MES proteins, the positive control (RFP), CmMES1.5-RFP, CmMES1.5-RFP, CmMES10.2-RFP, and CmMES17.3-RFP were introduced into *N. benthamiana* leaves by *Agrobacterium*-mediated transient transformation. Three days later, fluorescence of all four *MES* genes fused to RFP could be observed in the cytoplasm (**Figure 5**). The result indicated that the four MES proteins might function in the cytoplasm.

### 3.6 Functional analysis of the four *MES* genes associated with CBC-resistance

**Figure 6:**
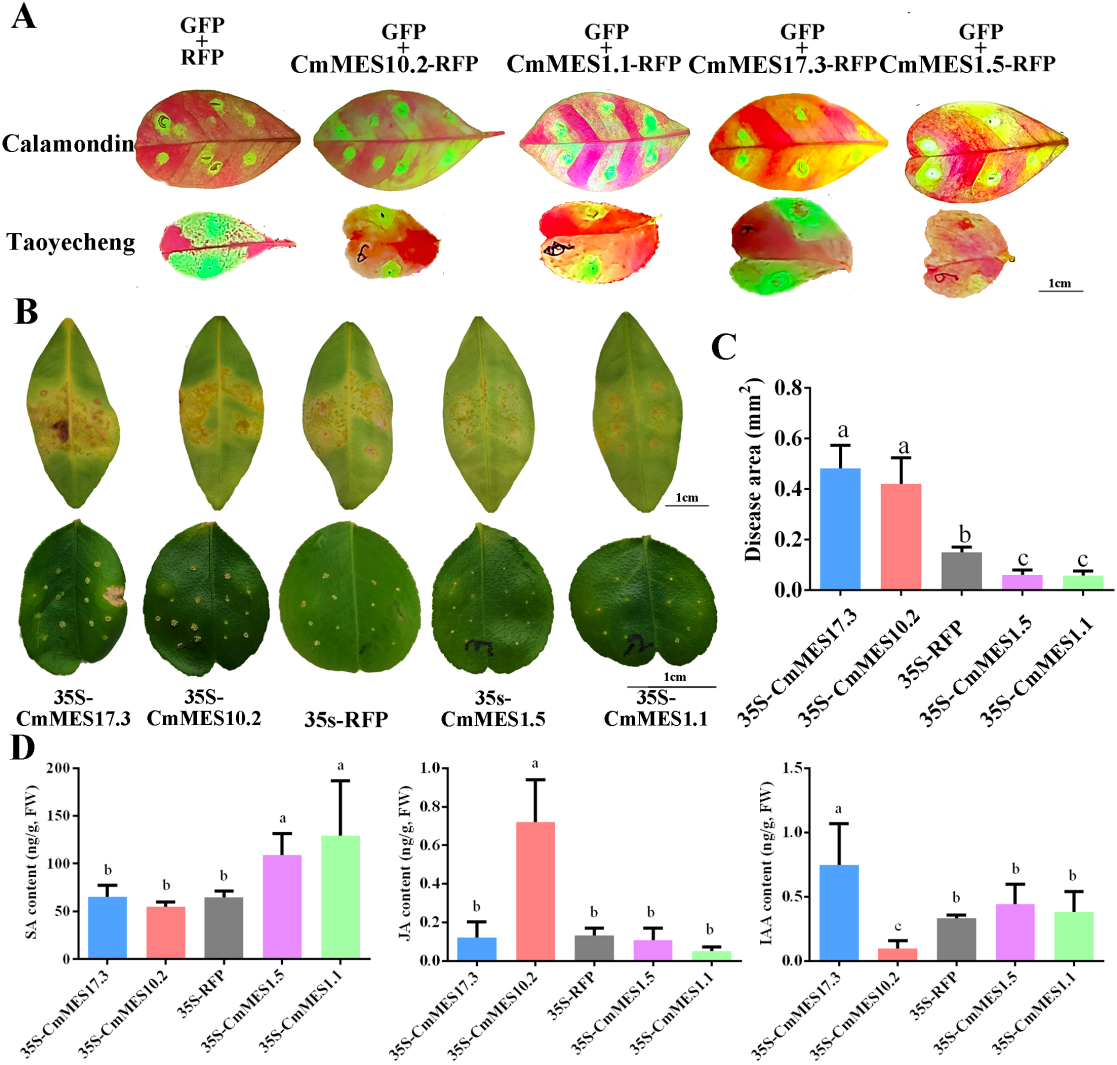
Functional identification of four citrus *MES* genes associated with CBC resistance. **(A)** Identification of transiently transgenic citrus leaves by green fluorescence observation. The overexpression vector contains two separate promoter regions. RFP is fused to the target gene for subcellular localization, and GFP is driven alone for fluorescence verification. **(B)** Citrus canker symptoms on transiently transgenic citrus leaves after *in vitro* infiltration inoculation with *Xcc*. The leaves above are calamondin leaves. 0.1mL 10^8^ cfu/ml of *Xcc* was injected into the leaves, and the water-soaked protrusions on the leaf surface were observed to evaluate CBC resistance. The leaves below are ‘Taoyecheng’ sweet orange. The CBC resistance was determined by dripping with 5 µL *Xcc* (10^8^ cfu/ml) to each puncture site made with a pin (0.5 mm in diameter), and the CBC resistance was evaluated by counting the lesion area. Scale bar = 1.0 cm. **(C)** Disease area on leaves of transiently transgenic citrus after *Xcc* inoculation (*p* < 0.05, ANOVAs with Tukey’s multiple range test). **(D)** Determination of SA, JA and IAA contents in calamondin leaves overexpressing four key MES genes. Different letters indicate significant differences (p < 0.05, ANOVAs with Tukey’s multiple range test). Data are the mean ± SD (*n*=3).

Transient overexpression in leaves of calamondin and ‘Taoyecheng’ sweet orange by infiltration were employed to verify the functions of the four *MES* genes. Green fluorescence observation showed that four key MES genes were successfully overexpressed in the leaves (**Figures 6A, B**). The quantitative analysis results showed that the expression levels of the four methylesterase genes were significantly increased in the transiently overexpressed citrus leaves compared with the control (**Supplementary Figures S4**). To identify the function of four key *MES* genes associated with CBC-resistance, transiently overexpressed leaves were *in vitro* inoculated with high concentrations (10^8^ cfu/ml) of *Xcc* suspension. The results of phenotypic observation and disease area statistics showed that overexpression of *CmMES17.3* and *CmMES10.2* increased the disease area, while overexpression of *CmMES1.1* and *CmMES1.5* decreased the disease area compared with the controls (**Figures 6B, C**). These results indicated that *CmMES17.3* and *CmMES10.2* might be negative regulatory factors for CBC resistance, while *CmMES1.1* and *CmMES1.5* might be positive regulatory factors.

The transiently overexpressed calamondin leaves were further employed to calculate the contents of SA, JA and IAA. The results showed that overexpression of *CmMES1.1* and *CmMES1.5* significantly increased the content of SA, and *CmMES17.3* increased the content of IAA, however, *CmMES10.2* increased the JA level (**Figure 6D**).

## 4 Discussion

CBC, as a major disease, seriously threatens the development of the citrus industry (Martins et al., 2020). Citrus endogenous hormones are important substances in response to CBC, especially SA, JA, and auxin. *MES* family genes participate in the demethylation pathway of MeSA, MeJA, and MeIAA, and produce active plant endogenous hormones to regulate plant resistance (Yang et al., 2008). In this study, we identified 13 *FcMES* genes which were divided into six groups in CBC-resistant kumquat. Among them, the *MES* genes in five groups were homologous *MES* genes of *Arabidopsis*, namely *AtMES1*, *AtMES10*, *AtMES11*, *AtMES12/14*, and *AtMES17*. In the meantime, *AtMES1*, *AtMES10*, *AtMES11*, *AtMES14*, and *AtMES17* also have homologous genes in grapes (Zhao et al., 2016). Furthermore, we demonstrated that CmMES1.1 and CmMES1.5 (homolog of AtMES1), CmMES10.2 (homolog of AtMES10) and CmMES17.3 (homolog of AtMES17) have a similar cytoplasmic localization as NtSABP2 (homolog of AtMES1) in tobacco and AtMES7 in *Arabidopsis* (Soares et al., 2022; Gao et al., 2021). These results indicated that the *MES* genes might have conserved functions during plant evolution.

Previous researches revealed that *MES* family genes played an important role in response to pathogen infection. For example, *GmSABP2-1* encodes methyl salicylate esterase and functions in soybean defense against soybean cyst nematode, and *Citrus sinensis CmMES1* play a positive role in the defense against CBC (Lin et al., 2024; Lima Silva et al., 2019). In this study, *MES1.1*, *MES1.5* and *MES10.2* were substantially upregulated at a later stage after *Xcc* infection in CBC-resistant varieties, but downregulated at a later stage after *Xcc* infection in CBC-susceptible varieties. *MES17.3* was down-regulated after *Xcc* infection, but the down-regulated level in CBC-resistant varieties was higher than that in CBC-susceptible varieties. These results implied that the expression of these *MES* genes might be associated with CBC resistance. In addition, the expression of the four *MES* genes was strongly activated or inhibited by high-concentration *Xcc* infection, but slightly activated or inhibited by low-concentration *Xcc* infection. Besides, the expression was strongly activated or inhibited at later stage (5D and 7D) upon *Xcc* infection. According to the symptomatic reactions, the leaves inoculated with high concentration *Xcc* showed severe water soaking at 5D, followed by hypersensitive necrosis at 7D. However, only slight water soaking was shown at the 7D after low concentration *Xcc* infection. In addition, *MES* family genes contain a large number of hormone response elements, such as ABRE which can be bound by abscisic acid responsive element (ABRE)-binding factor (ABF) (Han et al., 2024). Although these hormone response elements have been shown to respond to certain plant hormone treatments, the upstream regulatory mechanisms of many response elements are still unclear. Studies have shown that the content of ABA, SA, and JA will increase after *Xcc* infection, we hypothesized that the related transcription factors, such as ABFs would change with increasing plant hormone contents, which activate or inhibit the expression of *MES* family genes (Long et al., 2019). These results revealed that the expression of these *MES* genes might be associated with the development of CBC symptoms. Furthermore, these *MES* genes tended to be more highly expressed in young citrus tissues (including stems, leaves, and fruits) which were the major *Xcc* infection sites. Taken together, these *MES* genes might play crucial roles in CBC.

The *MES* family genes had catalytic activity for MeSA, MeJA, and MeIAA, and were usually important regulators of plant endogenous hormones in response to pathogen infection in plants (Vlot et al., 2008; Yang et al., 2008). In *A. thaliana*, AtMES1 mainly had the MeSA methylesterase activity, while the methylesterase activity of MeIAA and MeJA of AtMES1 was only 2% and 8%, respectively (Vlot et al., 2008). In sweet orange, the inhibitor of CsMES1 decreased the SA content and inhibited CBC resistance (Lima Silva et al., 2019). In this study, *CmMES1.1* and *CmMES1.5* were significantly up-regulated in CBC-resistant varieties but down-regulated in CBC-susceptible varieties at later stage after *Xcc* infection. Overexpression of *CmMES1.1* and *CmMES1.5* enhanced CBC resistance and significantly increased the SA content, but did not significantly increase IAA and JA content. Our results might explain why CBC-resistant varieties accumulated more SA than CBC-susceptible varieties after *Xcc* infection (Long et al., 2019). Therefore, *CmMES1.1* and *CmMES1.5* might be important genetic loci for CBC-resistant varieties to resist CBC. Furthermore, IAA could negatively regulate disease resistance by antagonizing the SA signaling pathway via JA (Xu et al., 2024). The previous study revealed that in the initial stage of citrus canker, exogenous NAA, an auxin analog, could significantly promote lesions formation (Cernadas and Benedetti, 2009). In *Arabidopsis*, AtMES17 had IAA methylesterase activity and could enhance the production of active IAA (Yang et al., 2008). In this study, the expression of *CmMES17.3* were substantially inhibited by *Xcc* infection. Overexpression of *CmMES17.3* promoted CBC susceptibility and increased the content of IAA. These results indicated that citrus might resist *Xcc* infection by inhibiting the expression of *CmMES17.3* to reduce the production of MeIAA methylesterase, hindering the conversion of inactive MeIAA into active IAA. In addition, JA could antagonize the function of SA, and negatively regulate plant resistance (Jiao et al., 2022; Huang et al., 2023). For example, JA could antagonize the function of SA to regulate *Arabidopsis* immunity and promote *Pseudomonas syringae* infection (Gupta et al., 2020). In this study, overexpression of *CmMES10.2* promoted CBC susceptibility and increased the content of JA. At present, only one *S* gene *LATERAL ORGAN BOUNDARIES 1* (*LOB1*) had been identified to be induced by *Xcc*, which enhanced CBC resistance after CRISPR gene editing and antisense oligonucleotide silencing (Hu et al., 2014; Jia et al., 2016; Peng et al., 2017; Su et al., 2023; de Lima et al., 2024). Therefore, *CmMES17.3* and *CmMES10.2* might provide alternative genes for gene editing to breed CBC-resistant varieties.

**Figure 7:**
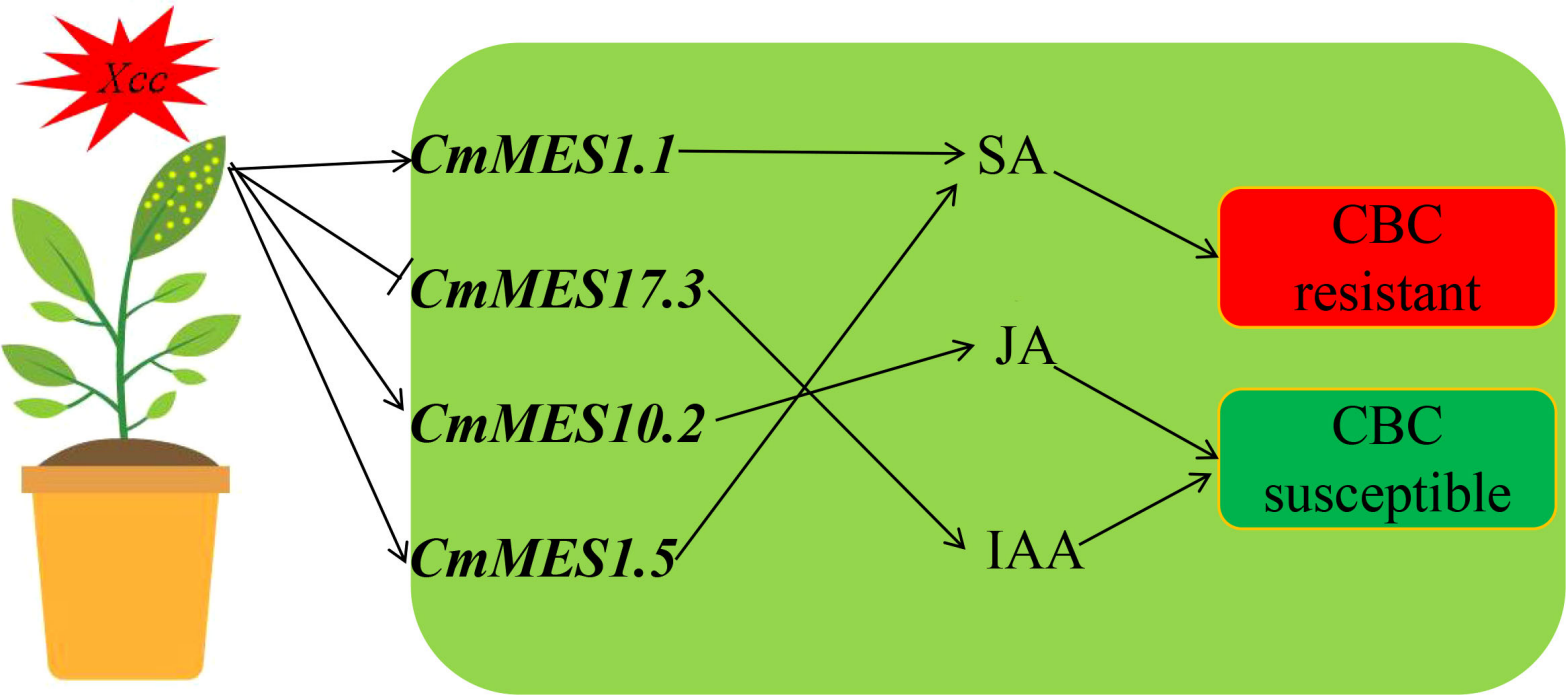
Model for *CmMES1.1*, *CmMES17.3*, *CmMES10.2*, and *CmMES1.5* regulating CBC resistance during infection with *Xcc*. After *Xcc* infection, the transcript levels of *CmMES1.1* and *CmMES1.5* in CBC-resistant varieties were significantly increased, increasing SA content, while the transcript level of *CmMES17.3* was significantly decreased, reducing IAA content, thereby enhancing canker resistance. The transcript level of *CmMES10.2* was also induced to increase, increasing the JA content, increasing the JA content and enhancing canker sensitivity.

Thus, a model was proposed to explain how *MESs* confer CBC resistance by manipulating plant endogenous hormone balance in citrus. *Xcc* infection might lead to increased expression of *CmMES1.1*, and *CmMES1.5*, which promoted SA production, and decreased expression of *CmMES17.3*, which inhibited IAA production (**Figure 7**).

## Data Availability

The datasets presented in this study can be found in online repositories. The names of the repository/repositories and accession number(s) can be found in the article/supplementary material.
