# Supplementary material for: Four MES genes from calamondin (Citrofortunella microcarpa) regulated citrus bacterial canker resistance through the plant hormone pathway
Source: Front Plant Sci. 2025 Jan 20;15:1513430. doi: 10.3389/fpls.2024.1513430 (PMC11788333; doi:10.3389/fpls.2024.1513430)
Supplement: Supplementary file 1 [file SupplementaryFile1.doc]

**Table S1 The transcriptome data of CBC-resistant ‘Meiwa’ kumquat (*F. crassifolia*) and CBC-susceptible ‘Mexican’ lime (*C. aurantifolia*) in response to *Xcc* infection at 24 h, 48 h and 72 h.**

| **Gene name** | **24h-C** | **24H-T** | **48h-C** | **48h-T** | **72h-C** | **72h-T** |
| --- | --- | --- | --- | --- | --- | --- |
| CaMES1.1 | 13.5365^*^ | 2.3451 | 29.1958 | 70.9571 | 57.5602 | 26.89187 |
| CaMES17.3 | 454.6674 | 513.5846 | 286.6499 | 38.4351 | 826.8451 | 270.9483 |
| CaMES10.2 | 110.6808 | 28.1416 | 167.2125 | 82.7833 | 127.5659 | 100.9714 |
| CaMES1.5 | 1367.9834 | 44.5576 | 6308.9526 | 19814.7677 | 6866.78135 | 1813.9325 |
| FcMES1.1 | 4.8542 | 12.5839 | 4.4303 | 103.8430 | 4.40370 | 81.2468 |
| FcMES17.3 | 1128.5931 | 67.1140 | 1355.6752 | 24.5944 | 1426.7960 | 53.2307 |
| FcMES10.2 | 0 | 23.0704 | 0 | 43.7234 | 0 | 459.4649 |
| FcMES1.5 | 4.8542 | 419.4624 | 4.4303 | 9302.1450 | 24.2203 | 38141.1906 |

^*^ FKPM (Fragments Per Kilobase Million) value

**Table S2 The transcriptome data of CBC-resistant calamondin in response to *Xcc* infection at 1 D, 3 D and 5 D.**

| **Gene name** | **1D-C** | **1D-T** | **3D-C** | **3D-T** | **5D-C** | **5D-T** |
| --- | --- | --- | --- | --- | --- | --- |
| CmMES1.1 | 22.8167^*^ | 57.2667 | 5.6667 | 12.26667 | 26.2067 | 129.3333 |
| CmMES1.4 | 6.1433 | 29.9800 | 97.9467 | 74.3733 | 95.0133 | 24.8100 |
| CmMES17.3 | 311.4900 | 175.3367 | 436.3433 | 288.8533 | 687.7667 | 81.5267 |
| CmMES17.1 | 11.2900 | 12.15337 | 22.9333 | 27.6100 | 5.6267 | 15.0767 |
| CmMES10.2 | 6.4000 | 12.6467 | 4.2600 | 8.1333 | 1.4267 | 8.8867 |
| CmMES1.5 | 73.1233 | 257.0900 | 15.8567 | 19.9533 | 58.7167 | 29.4333 |
| CmMES1.3 | 13.0533 | 14.9800 | 6.2833 | 5.1267 | 11.1667 | 1.6233 |
| CmMES11.5 | 12.0667 | 13.3567 | 6.8267 | 9.2300 | 7.1600 | 12.5667 |

^*^FKPM (Fragments Per Kilobase Million) value

**Table S3 Primers used in this study**

| **Gene name** | **Forward (5' to 3')** | **Reverse (5' to 3')** | **Function** |
| --- | --- | --- | --- |
| CmMES1.1 | ACAAATCAAATCCATCTCGCAT | CATCTGTAAATAGTAATCCTGGCC | qRT - PCR |
| CmMES1.5 | AATAAATCAAGTGCTTTCTCCGTTT | AGACAACAAAGTAATAAGCCCAAGC |
| CmMES10.2 | GTGGATCGAGTTTATGTCATCTGTG | TGGTTATCCTCGATCATCGCT |
| CmMES17.3 | CTGAACGTGACCGACGCC | GACATGTCAGCCGCAACGTA |
| CmMES1.1-RFP | ttacaattacgtcgacATGGAACCAACAGAGAAAGTTAAG | aggaggccatgtcgacT  TCAAATACCCTCTCCACAACATAA | Localization and Plant transform- ation vector |
| CmMES1.5-RFP | ttacaattacgtcgacATGGAAGAAGTAGTAGGCATGAA | aggaggccatgtcgacTGCATACTTAAGAGAAATCTGAGAC |
| CmMES10.2-RFP | ttacaattacgtcgacATGGAGGAGAGGAAGCATTTT | aggaggccatgtcgacTCTTCTTTGCAGATGACATAAACT |
| CmMES17.3-RFP | ttacaattacgtcgacATGCTGCTCCATAAAATCAGACAGT | aggaggccatgtcgacATTGATTGGAGCAGCTAGTTGAACT |

Note: Lowercase show vector adaptor

**Table S4 Accession numbers**

The genes sequences can download from the CPBD and NCBI database and the genes and their accession numbers are as follows:

| **Gene name** | Accession number | **Abbreviation** | Accession number |
| --- | --- | --- | --- |
| AtMES1 | AT2G23620.1 | FhMES1.1  CaMES1.1  FcMES1.1 | Sjg142100.1 |
| AtMES2 | AT2G23600.2 | FhMES1.2 | Sjg142100.2 |
| AtMES3 | AT2G23610.1 | FhMES1.3 | Sjg141660.1 |
| AtMES4 | AT2G23580.1 | FhMES1.4 | Sjg142150.1 |
| AtMES5 | AT5G10300.1 | FhMES1.5  CaMES1.5  FcMES1.5 | Sjg314100.1 |
| AtMES6 | AT2G23550.4 | FhMES10.1 | Sjg145040.1 |
| AtMES7 | AT2G23560.1 | FhMES10.2  CaMES10.2  FcMES10.2 | Sjg312980.1 |
| AtMES8 | AT2G23590.1 | FhMES11.1 | Sjg214930.1 |
| AtMES9 | AT4G37150.1 | FhMES11.2 | Sjg072760.3 |
| AtMES10 | AT3G50440.1 | FhMES11.3 | Sjg072760.2 |
| AtMES11 | AT3G29770.1 | FhMES11.4 | Sjg214930.2 |
| AtMES12 | AT4G09900.1 | FhMES11.5 | Sjg072760.1 |
| AtMES13 | AT1G26360.1 | FhMES14 | Sjg056470.1 |
| AtMES14 | AT1G33990.1 | FhMES17.1 | Sjg239980.1 |
| AtMES15 | AT1G69240.1 | FhMES17.2 | Sjg239980.2 |
| AtMES16 | AT4G16690.1 | FhMES17.3  CaMES17.3  FcMES17.3 | Sjg196610.1 |
| AtMES17 | AT3G10870.1 | Sjg190120.1 | Sjg190120.1 |
| AtMES18 | AT5G58310.1 |  |  |
| AtMES19 | AT2G23570.1 |  |  |
| AtMES20 | AT4G37140.1 |  |  |


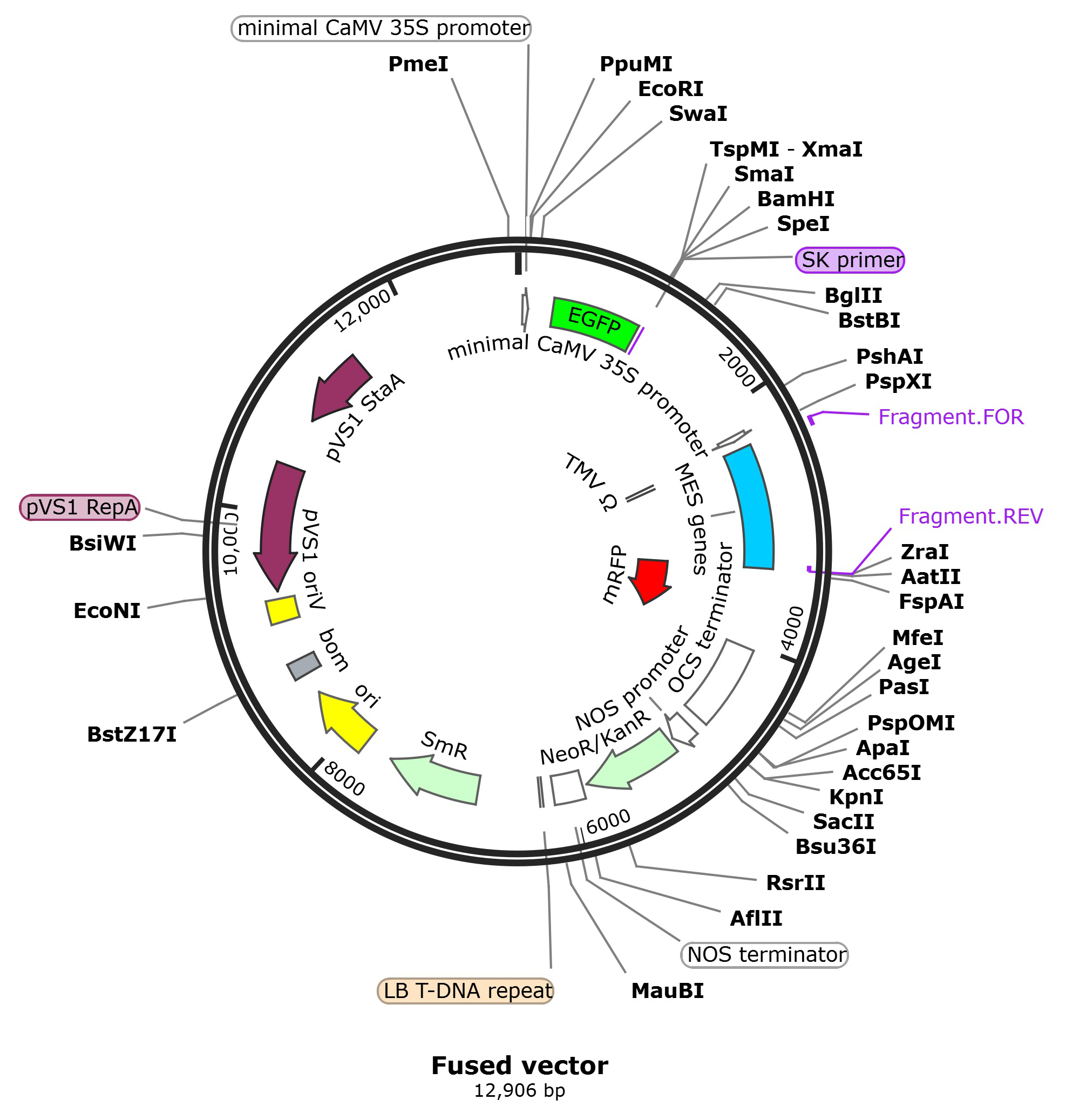


**Fig. S1 Overexpression vector map of *MES* family gene fusion with red fluorescent protein (mRFP)**

**
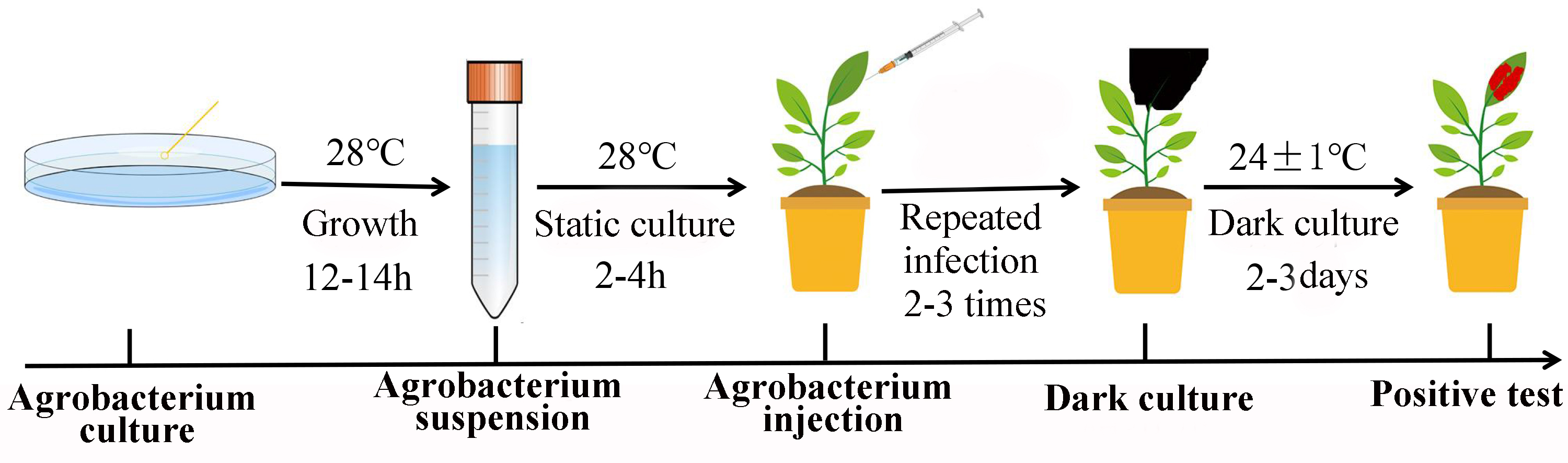
**

**Fig. S2 Improved transient transformation process of citrus leaves**


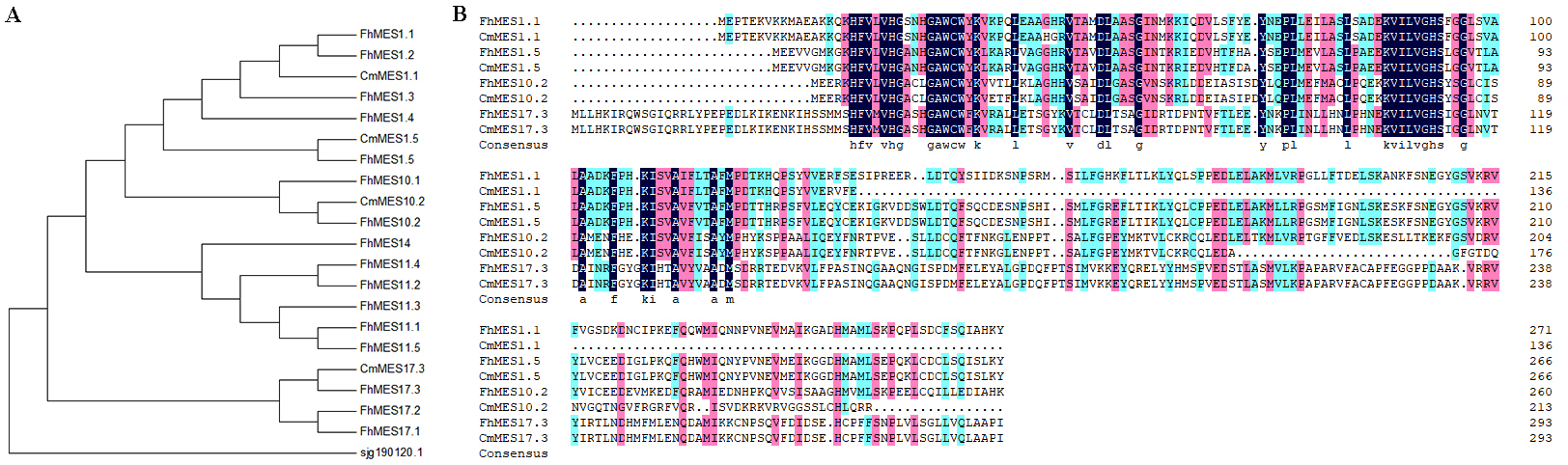


**Fig. S3 Phylogenetic analysis (A) and sequence alignment (B) of four *CmMES* genes.** The neighbor-joining tree and a bootstrap test with 1000 iterations were constructed using the MEGA7.


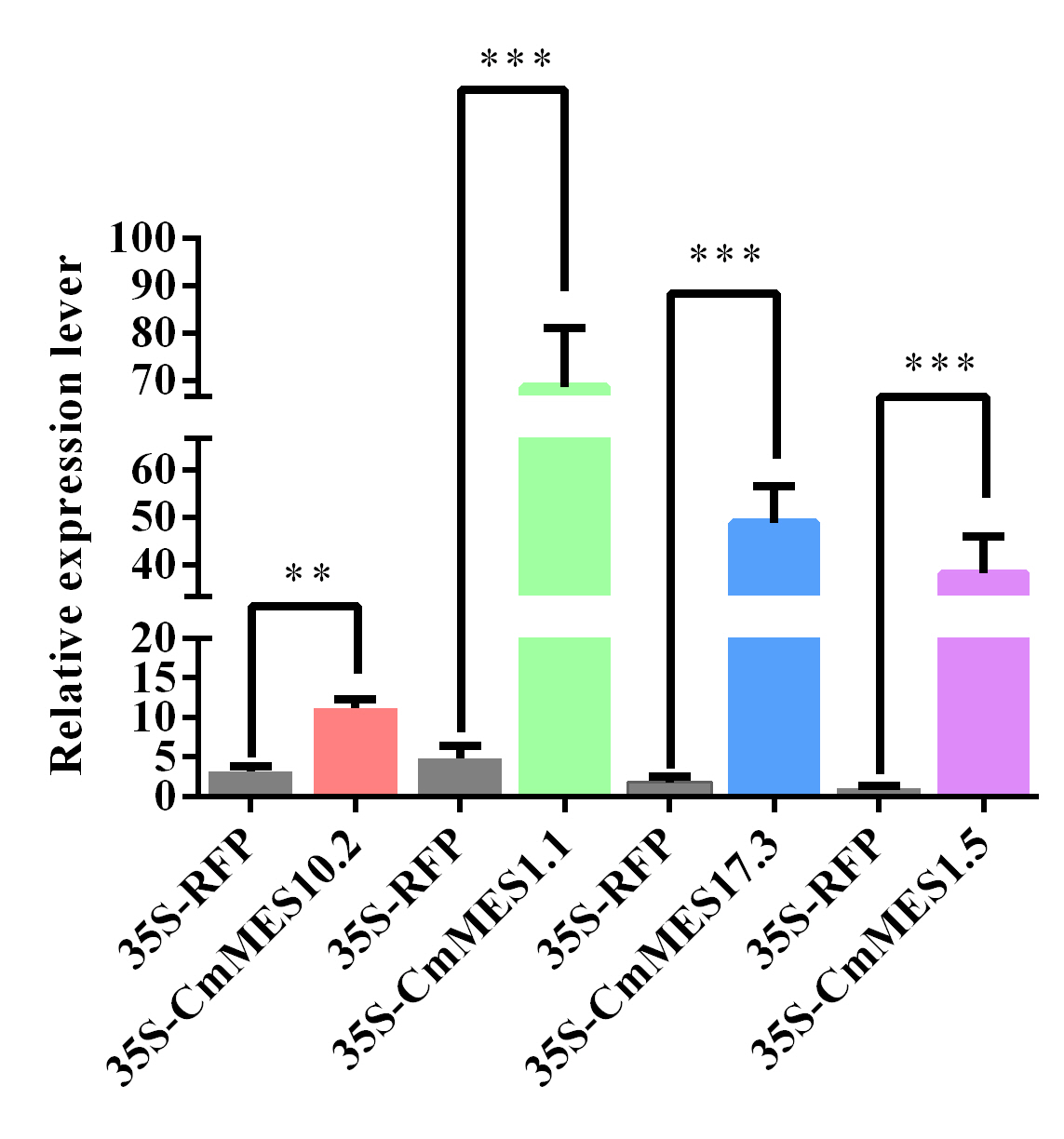


**Fig. S4 Relative expression level of four *MES* genes transient overexpression of citrus leaves.** Citrus leaves overexpression of RFP by CaMV 35S promoter is the blank control. *CmMES1.1*, *CmMES1.5*, *CmMES10.2* and *CmMES17.3* fusion RFP proteins were overexpressed by CaMV 35S promoter. Student’s t-test (***P*<0.01 and ****P*<0.001) was used for statistical analyses. Results are presented as means ±SD (*n*=3).
